# Early Molecular Stratification of High-risk Primary Biliary Cholangitis

**DOI:** 10.1016/j.ebiom.2016.11.021

**Published:** 2016-11-21

**Authors:** Claire Hardie, Kile Green, Laura Jopson, Ben Millar, Barbara Innes, Sarah Pagan, Dina Tiniakos, Jessica Dyson, Muzlifah Haniffa, Venetia Bigley, David E Jones, John Brain, Lucy J Walker

**Affiliations:** Institute of Cellular Medicine, Medical School, Newcastle University, Newcastle upon Tyne, UK

**Keywords:** PBC, UDCA, Prognosis, Stratification, NanoString® nCounter PanCancer Immunity Panel

## Abstract

High-risk primary biliary cholangitis (PBC), defined by inadequate response at one year to Ursodeoxycholic acid (UDCA), is associated with disease progression and liver transplantation. Stratifying high-risk patients early would facilitate improved approaches to care. Using long-term follow-up data to define risk at presentation, 6 high-risk PBC patients and 8 low-risk patients were identified from biopsy, transplant and biochemical archival records. Formalin-fixed paraffin-embedded (FFPE) liver biopsies taken at presentation were graded (Scheuer and Nakanuma scoring) and gene expression analysed using the NanoString® nCounter PanCancer Immunity 770-gene panel. Principle component analysis (PCA) demonstrated discrete gene expression clustering between controls and high- and low-risk PBC. High-risk PBC was characterised by up-regulation of genes linked to T-cell activation and apoptosis, INF-γ signalling and leukocyte migration and down-regulation of those linked to the complement pathway. *CDKN1a,* up-regulated in high-risk PBC, correlated with significantly increased expression of its gene product, the senescence marker p21^WAF1/Cip^, by biliary epithelial cells. Our findings suggest high- and low-risk PBC are biologically different from disease outset and senescence an early feature in high-risk disease. Identification of a high-risk ‘signal’ early from standard FFPE tissue sections has clear clinical utility allowing for patient stratification and second-line therapeutic intervention.

## Highlights

• 30% of PBC patients have high-risk disease defined after a year of non-response to ursodeoxycholic acid therapy.
• Gene-expression profiles from FFPE archival index liver biopsies demonstrate distinct clustering of high- and low-risk PBC.
• High-risk genes are linked to T-cell activation, apoptosis, migration; IFN-γ signalling; and biliary senescence.
• The risk ‘signal’, detectable from FFPE liver biopsy samples, has potential utility as a clinical diagnostic tool.

PBC is a chronic disease causing injury to bile ducts within the liver. 70% of patients have low-risk PBC and respond to treatment. The remaining 30% have un-responsive, high-risk disease that can progress to cirrhosis, liver-failure and the need for liver transplantation. Currently high-risk disease is only determined after a year of treatment failure. This study demonstrates that genes expressed in liver biopsies taken at diagnosis from patients with PBC allow high-risk disease to be identified earlier and this could be developed as a diagnostic tool. In addition, these data will guide further research and development of new treatments.

## 1 Introduction

Primary Biliary Cholangitis (PBC) is a chronic cholestatic liver disease with an autoimmune component to its aetiology. Pathogenesis is however complex with cholestatic injury to the small intra-hepatic bile ducts resulting from the actions of retained hydrophobic bile acids occurring in addition to direct immune injury (Dyson et al., 2015a). Importantly, PBC stratifies into two distinct clinical courses. For 70% of patients PBC is low-risk, non-progressive and responsive to ursodeoxycholic acid (UDCA) therapy, believed to act through reduction of secondary cholestatic injury. The remaining 30% have high-risk, progressive disease, unresponsive to UDCA and go on to develop fibrosis and ultimately biliary cirrhosis with its associated complications (Corpechot et al., 2011). High-risk patients are currently identified by non-response to UDCA at one year and whether the disease is biologically different from earlier stages of disease pre-treatment between high- and low-risk PBC has not previously been addressed. Early stratification of high-risk PBC is of clear clinical importance in establishing those likely to have progressive disease and to benefit most from novel second-line therapy.

Second-line therapies for PBC, and their integration into stratified approaches to management, are areas of current active research (Dyson et al., 2015b). Results have been variable, in particular in relation to the use of second-line immunomodulatory therapies. A potential reason for the apparent lack of efficacy of such therapies is the timing of their use (the challenge of “therapy sequencing” in a condition with both primary and secondary pathogenetic processes) (Dyson et al., 2015a). Need for second-line therapy being signalled by failure of response to a minimum of a year of UDCA therapy has clear limitations, with the risk of disease progression during that time. Importantly the use of immunomodulatory therapies (which work “upstream” in conventional models for the disease) is not considered until patients have failed anti-cholestatic therapy that targets “downstream” processes. The benefits of immunomodulatory therapy in PBC may therefore be missed, limited as much by our current timing of their use as by any intrinsic lack of efficacy.

A clinical tool that could allow for early identification of patients with high-risk PBC without the need to allow time for first-line therapy to prove itself ineffective would have clear utility. Current standard protocols for analysis of liver biopsy samples from patients with PBC do not allow for robust stratification early in the disease process. Formalin fixed paraffin embedded (FFPE) tissue remains the main-stay worldwide for diagnostic analysis of histomorphology and long-term preservation of tissue blocks but its use in molecular analysis has been hindered by difficulties of RNA degradation and the limitations of down-stream PCR-based techniques for RNA amplification. However, newer platforms of RNA analysis have opened up use of FFPE tissue for research and as a prospective molecular diagnostic tool (Mittempergher et al., 2011, Waldron et al., 2012), with even the possibility of reuse of tissue sections previously used for immunohistochemical staining (Al-Attas et al., 2016). The Nanostring® nCounter analysis system uses digital colour coded barcodes attached to single target specific probes corresponding to a gene of interest. Although a biased approach for the purposes of research, analysing a limited gene-set (up to 800 genes), the Nanostring® nCounter platform has clear advantages over other platforms for use in clinical settings. Sensitivity is comparable to qPCR (Geiss et al., 2008) and it is simple to use, highly automated, cost- and time- effective. RNA is measured directly with no amplification or other enzymatic processing and works well with degraded RNA such as that obtained from FFPE tissue (Reis et al., 2011) and compared to other RNA profiling platforms (Tyekucheva et al., 2015). This makes it a good candidate technology for the development of prognostic and diagnostic assays.

In this proof of concept study the Nanostring® nCounter 770 gene Pan-Cancer Immunity panel was used to compare gene-expression profiles in archival FFPE liver tissue obtained at the earliest point in the disease course from a historic PBC cohort with known disease outcome allowing stratification for future risk. The discovery of a distinct gene expression immune ‘signal’ at baseline between the two patient groups indicates high- and low-risk PBC are biologically distinct from early disease and points to potential treatment targets for high-risk PBC patients in the future. Use of FFPE tissue in this context has exciting implications for development of a new diagnostic and prognostic clinical tool, allowing better targeting of enhanced therapy.

## 2 Subjects and Methods

### 2.1 Study Design

The concept behind this study was to identify patients from a historic PBC cohort who had had a routine diagnostic liver biopsy performed and progressed to have either a positive outcome (UDCA response sustained over at least 15 years, alive and in full response at the study point (defined as low-risk patients)) or a poor outcome in terms of their PBC development (non-response to UDCA and progression leading to transplantation (defined as high-risk patients)), and to investigate any significant differences in the molecular characteristics of their liver tissue at the outset of disease (at a point where stratified second-line therapy to alter disease trajectory might be possible in the future). Transcriptomic profiling of archived formaldehyde-fixed paraffin embedded (FFPE) liver tissue was piloted using archive biopsy material from the pre-defined high and low risk patients. Ethical approval for this work is covered by the UK-PBC project (REC 14/NW/1146), a national cohort study.

### 2.2 Patient Groups

All study patients were female and aged between 36 and 66 at diagnosis of PBC. Diagnosis of PBC was determined by cholestatic liver biochemistry, presence of anti-mitochondrial antibodies (AMAs) which are present in up to 95% of patients and characteristic histological appearance of chronic, non-suppurative cholangitis that mainly affects the interlobular and septal bile ducts (Mittempergher et al., 2011). For the purposes of the study we used high stringency definitions of risk status. High-risk patients were formally defined for the study as follows:

1) Non-responders to treatment with UDCA at one year of treatment at a dose of 13–15 mg/Kg using Paris 1 criteria
2) Requiring liver transplantation for prognostic reasons for their PBC during subsequent follow-up.

Patients were excluded from this group if they had disease overlap with autoimmune hepatitis, histological evidence of other liver disease processes, or if their primary indication for liver transplantation was itch. These strict criteria assisted in ensuring homogeneity of the sample group and the minimisation of confounding factors during analysis. Low-risk patients were defined as follows:

1) Responsive to UDCA fully according to Paris 1 criteria.
2) Remained well and in full UDCA response after a minimum of 15 years follow up
3) Did not require liver transplantation.

Group allocation and meeting of study criteria were confirmed by an independent investigator. Time zero biopsy material from the donor liver of 3 liver transplant recipients was used for a non-disease comparator group.

Electronically recorded biopsy lists from 1992 to 2000 were reviewed in order to identify 96 low-risk patients. 46 patients were excluded as they had been discharged to their GP, limiting the accuracy of our follow-up records, 14 had clinical information suggesting UDCA under-response during follow-up, insufficient tissue was available for 20 patients and 8 were not recruited to the UK-PBC study precluding access to the tissue. This left 8 eligible patients in the low-risk category, all of whom were confirmed to be UDCA responders throughout their disease course and had a liver biopsy taken at the point of presentation with the condition.

49 potential high-risk patients were identified from the transplant recipient list. Two were excluded due to itch as the primary indication for transplantation, four were excluded for cross-over with other autoimmune liver diseases, 33 due to insufficient tissue available and three were not recruited to UK-PBC. This left eight patients for inclusion in the study as high-risk participants. All patients were Caucasian females aged between 39 and 55 years at the time of their first diagnostic biopsy.

### 2.3 Tissue

FFPE liver tissue samples were obtained from the cellular pathology department archive of Newcastle Hospitals NHS Foundation Trust. 10 μm curls were cut from the FFPE blocks, discarding the outer 40 μm. Liver tissue examined included blocks of core liver biopsies from patients with PBC and time zero, non-disease liver biopsies.

### 2.4 RNA Purification

mRNA was extracted from 10 μm FFPE curls using AllPrep kit (Qiagen, Hilden, Germany). 320 μl of de-paraffinisation solution (Qiagen) was used for each sample then manufacturer's instructions were followed to isolate the ribonucleic acid (RNA). This kit was chosen as it has been reported to be the most appropriate for RNA isolation from FFPE tissue (Al-Attas et al., 2016) as it enables reversal of modification of RNA by the formaldehyde preservation process. mRNA was assessed for quantity and purity using a NanoDrop spectrophotometer (NanoDrop ND-1000, Thermo Scientific, Wilmington, USA). An Agilent Bioanalyzer RNA assay kit (GCB, Durham, USA) was used to measure the RNA integrity of a number of the samples. Comparison between quality and age of sample was carried out to assess suitability of old tissue.

### 2.5 RNA Array

The nCounter Analysis System (NanoString®, Seattle, USA) was used to analyse the gene expression levels in the mRNA extract, first using the 18-gene Human Reference panel, then the 770-gene PanCancer Immune Profiling panel. The system works by solution-based mRNA hybridisation of short length (50mer) probes, which are subsequently fixed to a biotin-coated cartridge. This is then digitally imaged and counted to quantify expression. GEO data available at: http://www.ncbi.nlm.nih.gov/geo/query/acc.cgi?acc=GSE79850. Accession GSE79850.

### 2.6 Pathological Scoring

The scoring of pathological specimens was simultaneously performed by two pathologists (JGB and DGT) using H&E, Orcein and Sirius red fast green histological stains. Biopsies were assessed using hepatic activity index parameters for interface hepatitis (none, mild, moderate or severe), portal inflammation (none, mild, moderate or severe). The presence of ductopenia (> 66% of portal tracts without a bile duct), Scheuer stage (1–4) and the components of the Nakanuma score were given (CA0-CA3 and HA0-HA3) rather than the summative Nakanuma stage.

### 2.7 Immunohistochemical (IHC) Staining of Paraffin Embedded Liver Tissue

Anonymised FFPE sections were de-waxed in xylene for five minutes then rehydrated through graded alcohols and washed in tap water. Sections were then treated in 0.2% hydrogen peroxide (H_2_O_2_) block for ten minutes before being washed in tap water. Sections were subsequently treated in boiling citrate buffer for two minutes in a pressure cooker and washed in tap water. Blocking was carried out using anti-rabbit/goat IgG Vectastain ABC kit (Vector, Peterborough, UK) for ten minutes then, to reduce endogenous biotin, an avidin-biotin blocking kit (Vector) was used. Blocking serum was replaced with primary antibody (p21^WAF1/Cip^ Santa Cruz Biotechnology #CatSc-6246; RRID:AB_628073) diluted in tris-buffered saline (TBS) pH 7.6 for one hour and replaced with just TBS on negative controls. Secondary and tertiary antibodies anti-rabbit/goat IgG Vectastain ABC kit (Vector Laboratories Cat #PK-7100 RRID:2336827) were used to develop the sections according to manufacturer's instructions.

3′ 3′ diaminobenzidine tetrehydrochloride (DAB) was used to develop the sections. Mayers haematoxylin was used to counterstain, sections were washed in tap water then Scott's tap water substitute. Sections were washed in tap water then dehydrated through graded alcohols and mounted in DPX. This technique was used to examine differences in expression in the liver of p21 in high and low risk disease. To ensure objectivity, two observers (BM and BI) were trained by JB to interpret immunohistological staining for p21. Scoring was then performed in a blind fashion by the assessment of number of positive biliary epithelial cells per 10 hpf, and corroborated by JB.

## 3 Statistical Analysis

Statistical analysis of Nanostring® nCounter mRNA expression data was performed using a custom analysis pipeline based on the ‘R’ programming language, two-tailed *t*-test was used as recommended by manufacturer with significant genes to p < 0.05 with fold change > 1.5 after Benjamini-Hochberg false discovery rate correction. Gene expression patterns were assessed using String (Jensen et al., 2009). This data was assessed using principle component analysis (PCA) where genes are weighted by the according to the sample variance they account for. GraphPad Prism 6.0 was used for immunohistochemistry statistical analysis. Pathway analysis was performed using GeneMANIA analysis software (Warde-Farley et al., 2010).

## 4 Results

### 4.1 Sample Selection and Patient Stratification

RNA analysis of first liver biopsies was performed for PBC patients with defined subsequent clinical outcomes. Suitable cohorts (high- and low-risk defined using stringent criteria) were identified by reviewing our clinical database. High-risk patients were defined as non-responders to treatment with UDCA at 1 year using Paris 1 criteria and subsequently requiring liver transplantation. Low-risk patients were defined as responders to UDCA at one year and still responsive after a minimum of 15 years follow up. Patient details are given in Table 1. Time zero biopsies taken from normal liver during transplantation were used as non-disease controls. Six high-risk patients, eight low-risk patients and three controls were identified. Histological staging of the biopsy material is found in Table 2.

### 4.2 RNA Extraction From FFPE Samples

RNA was successfully extracted from formalin fixed paraffin embedded (FFPE) tissue from pre-treatment, diagnostic liver biopsies and was of sufficient quality for transcriptomics using the PanCancer Immune Profiling panel from NanoString Technologies®.

### 4.3 Identification of Genes Differentially Expressed Between Low- and High-risk PBC

**Fig. 1:**
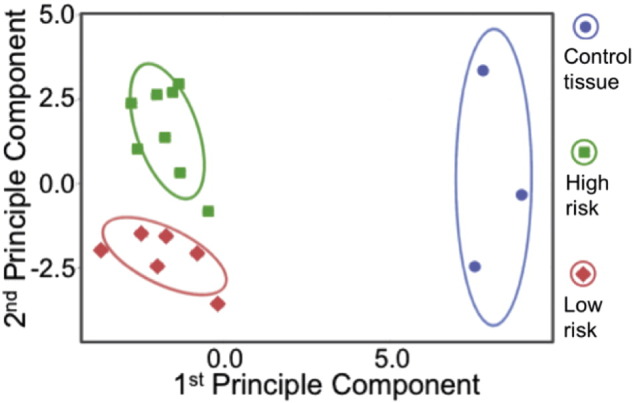
Principle component analysis (PCA) for transcriptional signatures in low-risk and high-risk early PBC. PCA displays sample clustering (low-risk PBC, high-risk PBC and controls) following identification of the top 57 differentially expressed genes by two-tailed *t*-test.

Gene expression data were analysed according to the manufacturer's instructions and showed 107 immune-related genes to be significantly different between high- and low-risk disease (Table 3). Principle component analysis (PCA) was employed to display the top 57 differentially expressed genes identified after the two-tailed *t*-test. This analysis was able to cluster samples discretely by PBC risk group, (Fig. 1), with both groups also having a distinct clustering compared to control liver.

**Fig. 2:**
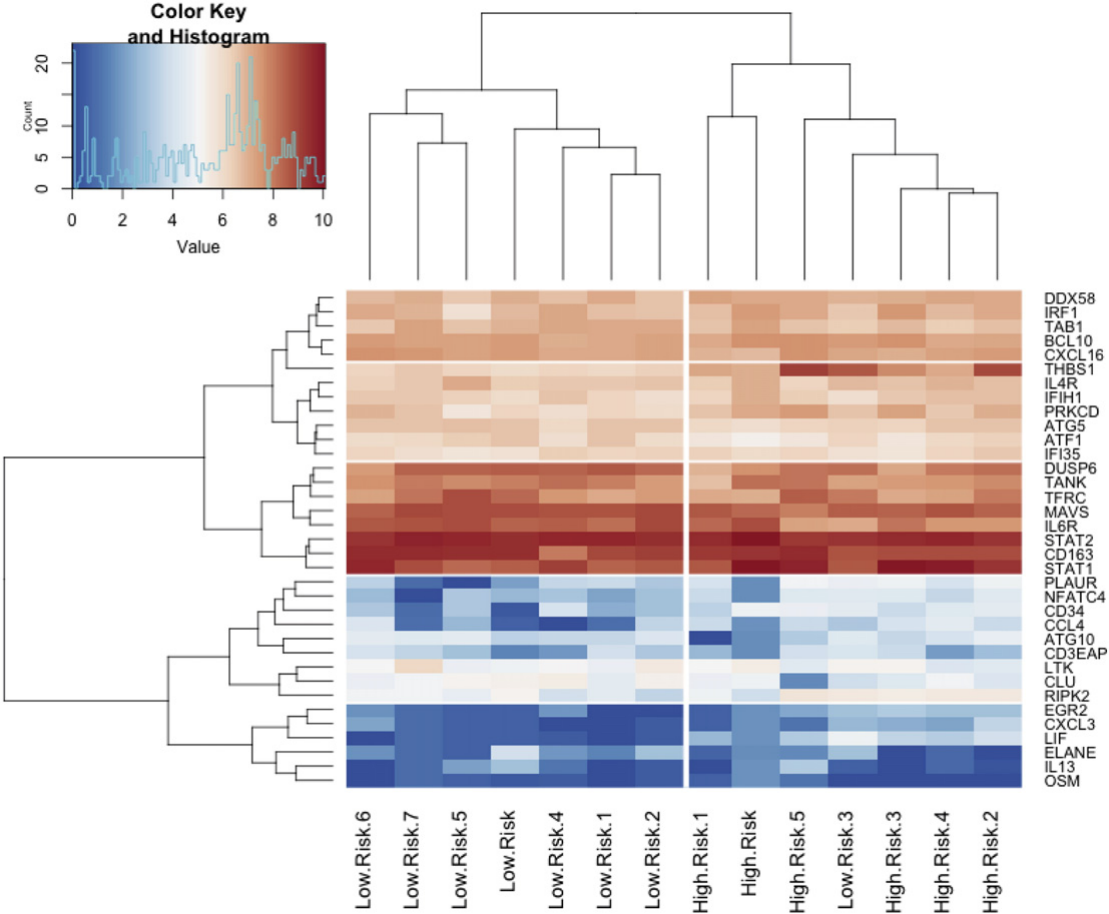
Dendrogram of genes identified by PCA to most separate high- and low-risk PBC groups. Dendrogram generated from the 34 genes identified by *t*-test to most separate high- and low-risk PBC. Colours indicate scale of gene expression (red: up-regulation; blue: down-regulation).

**Fig. 3:**
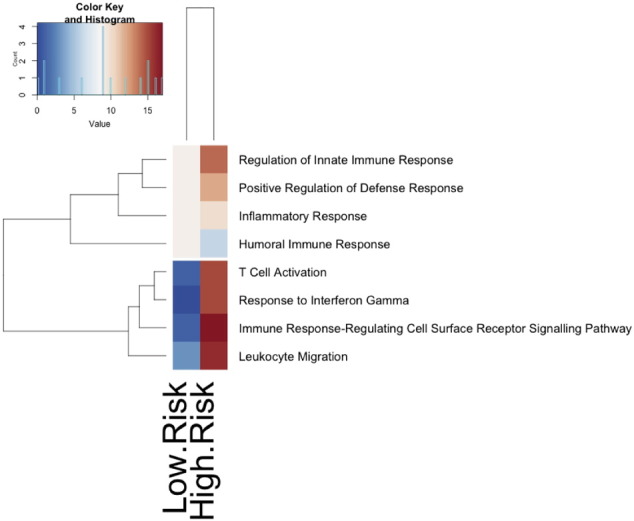
Pathway analysis and heatmap for high- and low-risk PBC.Pathway analysis of the top 8 pathways of differentially expressed genes for high- and low-risk PBC (red: up-regulation; blue: down-regulation).

A dendrogram (Fig. 2) using 34 genes identified by *t*-test that most separated the high-risk from the low-risk PBC groups was generated to visualise the sample. Only one low-risk biopsy was grouped with the high-risk strata and there were no high-risk biopsies in the low-risk strata. Interestingly, patients with high-risk disease were characterised by up-regulation of genes linked in particular to apoptosis and cell cytotoxicity as well as down regulation of the complement pathway in comparison to low risk and control tissue. Pathway analysis of the top 8 pathways of differentially expressed genes found T cell activation, IFNγ response, T-cell activation and leukocyte migration to be most significant in high-risk disease (Fig. 3).

**Fig. 4:**
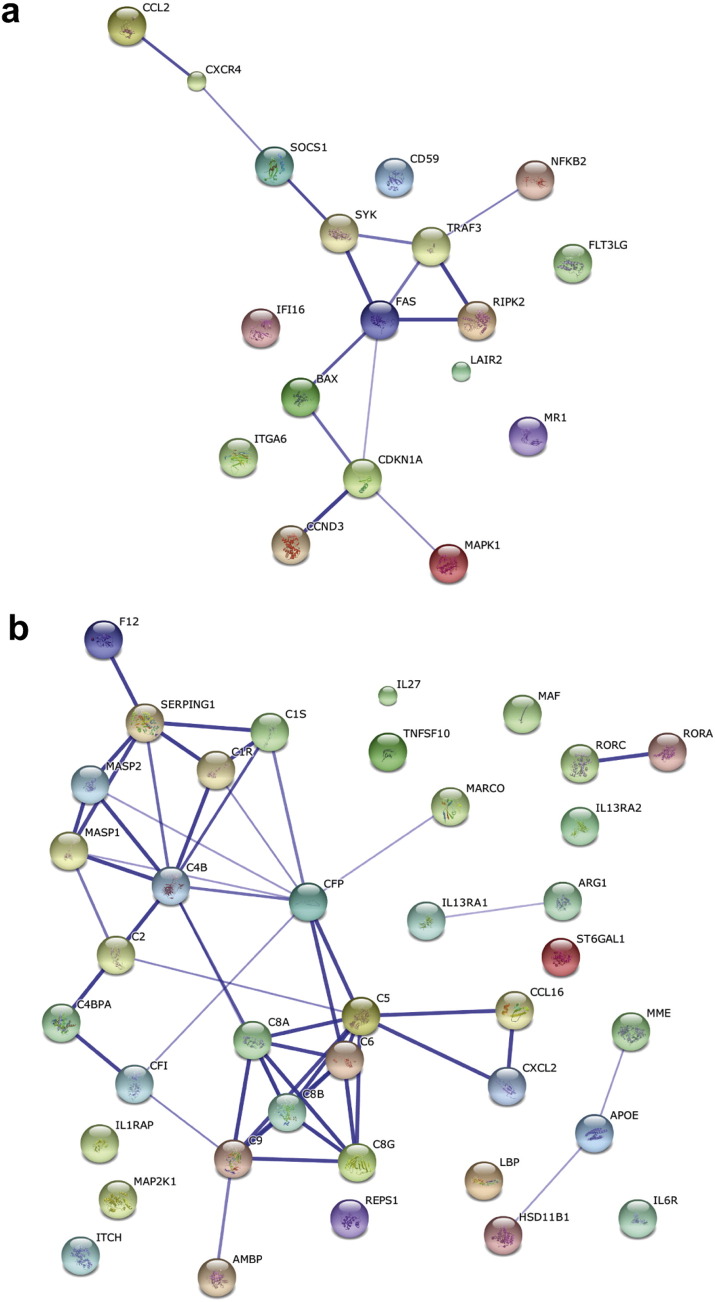
GeneMANIA pathway analysis of up-regulated genes in high- and low-risk PBC. Diagrams show GeneMANIA linked up-regulated gene-products that participate within the same biological pathway in (a) high-risk PBC and (b) low-risk PBC.

Differentially expressed genes were entered into geneMANIA to further clarify the pathways contributing to the difference between high- and low-risk patients (Fig. 4). As anticipated, pathways related to interferon-γ response, leukocyte migration, T cell activation and apoptosis were the most significant in high-risk disease.

### 4.4 p21^WAF1/Clp^ Expression Is Associated With High-risk PBC and CDKN1a mRNA Expression Levels

**Fig. 5:**
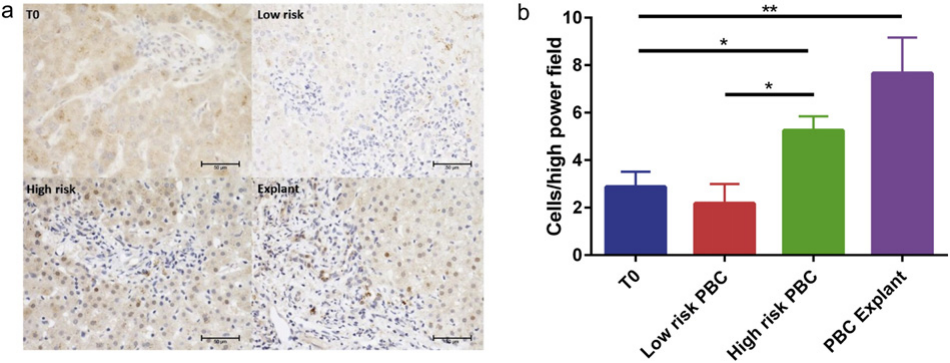
p21^WAF1/Clp^ expression by biliary epithelial cells from time-zero, low-risk PBC, high-risk PBC and explanted liver biopsies. (a) Representative images generated by immunohistochemistry showing p21^WAF1/Clp^ expression by biliary epithelial cells from time-zero (T0), high-risk PBC, low-risk PBC and explanted liver biopsies. (b) Cumulative data of p21^WAF1/Clp^ expression by biliary epithelial cells from T0, high-risk PBC, low-risk PBC and explanted liver biopsies. One-way ANOVA (*p < 0.05, **p < 0.001).

The gene product of *CDKN1a* (which was up-regulated with a 1.78 fold change) (p = 0.032) in expression in high- compared to low-risk PBC (Table 3), p21^WAF1/Cip^, is a marker of biliary senescence and has been shown to predict outcome in a number of liver diseases and to correlate with Scheuer stage (Sasaki et al., 2010). Biliary epithelial cell staining for p21^WAF1/Cip^ by immunohistochemistry (Fig. 5) shows significantly higher expression levels in explanted tissue versus time zero biopsies and also on bile ducts in high- compared to low-risk patients and controls.

## 5 Discussion

This study demonstrates high-risk PBC to be fundamentally biologically distinct at an early stage from more indolent forms of the disease. PBC risk is therefore pre-determined and not defined by UDCA response, as is the current model of stratification. Risk of progression could be predicted from the baseline liver mRNA expression ‘signal’ obtained from conventionally processed FFPE liver tissue, allowing for potential development of an early clinical stratification tool to inform not only patient management and follow up but also the development and early use of novel second-line therapies (Dyson et al., 2015a); an important issue given recent data highlighting the rapid nature of PBC progression (Carbone et al., 2016).

Current histological methods of scoring lack sensitivity in differentiating high- and low-risk PBC patients in early disease. In this study, patients with high-risk disease tended to have a higher Scheuer score at their first biopsy, although there was no clear-cut off point. The cholangitis component of the Nakanuma score (Carbone et al., 2013, Thurairajah et al., 2013) appeared to enable a degree of differentiation between high- and low-risk disease, but the hepatitis component did not. It was notable, however, that all patients with high-risk disease had evidence of ductopenia on biopsy, only present in one low risk patient. Further confirmation of ‘high-risk’ features using molecular techniques has a clear role in disease stratification.

Although using archival FFPE liver tissue, we were able to demonstrate that mRNA could be successfully extracted for analysis using the Nanostring® nCounter platform from FFPE blocks dating back over 20 years. RNA expression-signatures from FFPE tissue has previously been difficult to assess due to high levels of degradation (von Ahlfen et al., 2007, Thompson et al., 2013). Despite patient sample sizes being small, the significantly different molecular signature demonstrated by PCA and hierarchical clustering differentiating high- and low-risk disease is an important finding. Although limited in scope and requiring validation in further patient cohorts, this study offers real potential for the development of a molecular ‘signature’ gene set for use as a practical therapy stratification tool essential for targeted intervention. Advantages of the Nanostring® nCounter platform in this setting are clear. No modifications to standard pathology handling of tissue samples would be required and data suggests a reference-based strategy, applicable to single-patient molecular testing, would allow for correction of the systemic and technical variations in measurements that lead to the batch effect inherent in the handling of such samples (Talhouk et al., 2016).

In terms of pathogenesis, we have demonstrated clear differences in both the ‘immune’ and ‘senescence’ phenotype between high- and low-risk PBC from early stages of disease. What we have likely identified is two distinct disease processes. Low-risk disease appears to be characterised by cholestatic biliary injury in the absence of immune-mediated damage and therefore treatable by the detoxifying effect of UDCA. High-risk disease, however, is characterised by T cell activation and apoptosis causing on-going bile-duct damage un-modifiable by UDCA alone. Up-regulation of *CDKN1a*/p21^WAF1/Cip^ expression by biliary epithelial cells in early disease not only identifies a potential risk marker for use in early disease stratification, but indicates biliary senescence to be an early and differentiating process in high-risk PBC. Furthermore, this study serves to validate data from genome-wide association studies (GWAS) of un-stratified PBC cohorts highlighting the importance of immune pathways in PBC pathogenesis (Mells et al., 2011, Cordell et al., 2015). Nine of the genes with significantly up-regulated expression identified by this study in high-risk disease correspond to candidate genes identified in PBC GWAS studies (*HLA-DQ1B, SOCS1, CD80, TNFSF15, HLA-G, HLA-A, HLA-B, IL-18, STAT1*) indicating important pathways for further study. An immune directed RNA panel was used as published genome-wide association data suggested associated immune pathways to be the most relevant to disease progression (Mells et al., 2011), however unbiased methods of transcriptomic analysis may provide greater information for on-going research and a prospective unbiased transcriptomic analysis of PBC patient biopsies is required.

Although data presented here are essentially descriptive and require pro- and retrospective validation in larger cohorts, the potential for identifying high-risk patients at an early stage is clear, enabling stratified intervention as novel therapeutics come to trial. Early stratification would also be of benefit in triaging low-risk patients, allowing follow up in primary care without need for expensive specialist resources. With support rising for centralised molecular pathology institutions, concurrent transcriptomics represents a feasible method to determine patient management in conjunction with current histopathological diagnostic, grading and staging approaches.

## Funding Sources

Overall the project was funded by the PBC-UK MRC Stratified Medicine grant (MR/L001489/1). L.J.W. and J.B. are funded by the NIHR Clinical Lectureship programme. L.J.W. is also supported by a Starter Grant for Clinical Lecturers funded by the Academy of Medical Sciences (SGL009/1013). V.B. and M.H. are supported by Wellcome Trust intermediate (WTX022255) and senior (WT107931/Z/15/Z) fellowship programmes respectively.

## Conflicts of Interest

D.E.J. works as a consultant for Intercept, GSK, Pfizer, Novartis and has grant funding from Intercept. J.B. is supported by grant funding from Intercept (BH124127), GSK (BH112262) and Pfizer (BH152715).

## Author Contributions

**Table:** 

Author name	Affiliation	Contact	Contribution
Claire Hardie	Newcastle University	clairemhardie@gmail.com	Acquisition of data, interpretation of data, drafting of manuscript.
Kile Green	Newcastle University	kile.green@ncl.ac.uk	Acquisition of data, statistical analysis, interpretation of data, technical support.
Laura Jopson	Newcastle University	Laura.jopson@doctors.org.uk	Study design and acquisition of data.
Ben Millar	Newcastle University	ben.millar@ncl.ac.uk	Acquisition of data, interpretation of data.
Barbara Innes	Newcastle University	barbara.innes@ncl.ac.uk	Acquisition of data, technical support.
Sarah Pagan	Newcastle University	sarah.pagan@ncl.ac.uk	Technical support, study design.
Dina Tiniakos	Newcastle University	Dina.tiniakos@ncl.ac.uk	Blinded staging of liver biopsies, interpretation of data.
Jessica Dyson	Newcastle University	jessicadyson@doctors.org.uk	Study supervision, drafting of manuscript.
Venetia Bigley	Newcastle University	Venetia.bigley@ncl.ac.uk	Study supervision and technical support.
Muzlifah Haniffa	Newcastle University	m.a.haniffa@ncl.ac.uk	Study concept.
David Jones	Newcastle University	david.jones@ncl.ac.uk	Study concept, design and supervision, interpretation of data, drafting of manuscript, obtaining funding.
John Brain	Newcastle University	john.brain@ncl.ac.uk	Study composition, study supervision, blinded staging of liver biopsies, interpretation of data, drafting of manuscript.
Lucy Walker	Newcastle University	lucy.walker2@ncl.ac.uk	Study concept, design and supervision, drafting of manuscript.

## Figures and Tables

**Table 1:** Patient characteristics.

Baseline characteristics	High-risk PBC	Low risk PBC
Sex, *n (%)*		
Female	6 (6)	8 (8)

Ethnic group, *n (%)*		
White, British	6 (6)	8 (8)
White, Other	0 (6)	0 (8)

Age, *y*		
Mean (SD)	45 (6.46)	57 (8.32)
Range	36–55	43–66

Laboratory markers, *mean (SD)*		
ALP, U/L	696.6 (432.7)	302.6 (201.6)
Bilirubin, mg/dL	48.2 (18.6)	8.75 (2.63)
Albumin, g/dL	40 (2.12)	51.5 (18.6)
Platelets, 10^3^/μL	218.33 (105.3)	366 (67.9)

PBC inclusion criteria, *n (%)*		
Increased ALP	6 (6)	8(8)
Positive AMA titre	5 (6)	8 (8)

Positive ANA titre		
Liver biopsy	6 (6)	8 (8)

UDCA response (Paris 1)		
Yes	0 (6)	8 (8)
No	6 (6)	0 (8)

**Table 2:** Biopsy scoring.

Category	Scheuer stage	Interface hepatitis	Portal inflammation	Nakanuma score	Ductopenia
High risk	4	Moderate	Moderate	CA2 HA1	Yes
High risk	3	Mild	Moderate	CA3 HA1	Yes
High risk	4	Moderate	Moderate	CA1 HA3	Yes
High risk	3	Mild	Moderate	CA1 HA3	Yes
High risk	4	Moderate	Moderate	CA1 HA2	Yes
High risk	3	Mild	Mild	CA1 HA1	Yes
Low risk	1	None	Mild	CA1 HA0	No
Low risk	1	None	Mild	CA0 HA1	No
Low risk	1	None	Mild	CA2 HA2	Yes
Low risk	1	None	Mild	CA1 HA0	No
Low risk	2	Moderate	Moderate	CA2 HA1	No
Low risk	2	None	Mild	CA1 HA2	No
Low risk	1	None	Mild	CA1 HA1	No
Low risk	1	Mild	Moderate	CA2 HA2	No
Control	0	None	None	NA	No
Control	0	None	None	NA	No
Control	0	None	None	NA	No

**Table 3:** Genes with A significant upregulation in early high risk disease and B significant upregulation in early low risk PBC. Two-tailed t-test was used as recommended by manufacturer, and genes shown are significant to p < 0.05 with fold change > 1.5 after Benjamini and Hochberg correction.

Gene	Name	p-Value	Fold change
A Increased expression in high-risk PBC			
HLA-DQB1	Major Histocompatability Complex, Class 2, DQ beta 1	0.017	13.57
SOCS1	Suppressor of Cytokine Signalling 1	0.009	5.67
CD24	CD24 Molecule	0.028	5.39
SLAMF1	Signalling Lymphocytic Activation Molecule Family Member 1	0.026	4.99
CARD11	Caspase Recruitment Domain Family Member 11	0.040	4.46
LY86	Lymphocyte Antigen 86	0.040	4.36
COL3A2	Collagen, type III, alpha 2	0.010	4.11
MFGE8	Milk Fat Globule-EGF Factor 8 Protein	0.006	4
CD80	CD80 Molecule	0.049	3.8
FCER2	Fc Fragment of IgE Receptor II	0.033	3.77
CCL3L1	Chemokine (C-C motif) Ligand 3 Like 1	0.014	3.75
ISG20	Interferon-Stimulated Gene 20 kDa Protein	0.017	3.73
TNFSF15	Tumor Necrosis Factor Superfamily Member 15	0.038	3.61
LAIR2	Leukocyte Associated Immunoglobulin Like Receptor 2	0.003	3.22
MCAM	Melanoma Cell Adhesion Molecule	0.011	3.17
HLA-G	Histocompatibility antigen, class I, G	0.030	3.12
S100B	S100 calcium-binding protein B	0.038	3.08
IRF4	Interferon Regulatory Factor 4	0.044	3.06
CXCR4	C-X-C Motif Chemokine Receptor 4	0.018	3.03
CCL4	C-C Motif Chemokine Ligand 4	0.046	3.01
CXCR3	C-X-C Motif Chemokine Receptor 3	0.024	2.99
CD34	CD34 Molecule	0.017	2.97
ITGA6	Integrin alpha-6	0.019	2.87
BCL2	B-cell lymphoma 2	0.039	2.82
TNFRSF11B	Tumor Necrosis Factor Receptor Superfamily Member 11b	0.015	2.7
RUNX1	Runt-related transcription factor 1	0.032	2.58
KLRC1	Killer Cell Lectin Like Receptor *C*1	0.045	2.57
COL3A1	Collagen, type III, alpha 1	0.024	2.5
TLR6	Toll-like Receptor 6	0.025	2.47
CD83	CD83 Molecule	0.011	2.41
SPN	Sialophorin	0.050	2.36
ITGAX	Integrin, alpha X (complement component 3 receptor 4 subunit)	0.005	2.31
NLRC5	NOD-like receptor family CARD domain containing 5	0.047	2.15
RELB	RELB Proto-Oncogene, NF-KB subunit	0.015	2.1
ADA	Adenosine deaminase	0.004	2.08
IL18	Interleukin-18	0.050	2.08
CCL13	Chemokine (C-C motif) Ligand 3 Like 13	0.035	2.04
IFI16	Gamma-interferon-inducible protein 16	0.012	2
AMICA1	Adhesion Molecule Interacting with CXADR Antigen 1	0.035	1.99
PNMA1	Paraneoplastic Ma Antigen 1	0.049	1.97
SYK	Spleen Tyrosine Kinase	0.017	1.87
TAP1	Transporter1, ATP-Binding Cassette, Sub-Family B	0.034	1.87
ABCB1	ATP-Binding Cassette Subfamily B Member 1	0.008	1.79
CDKN1A	Cyclin-Dependent Kinase Inhibitor 1A	0.032	1.78
STAT1	Signal Transducer and Activator of Transcription 1	0.015	1.76
NFKB2	Nuclear Factor NF-kappa-B p100 Subunit	0.020	1.75
CCND3	Cyclin D3	0.042	1.74
PRKCE	Protein Kinase C Epsilon	0.042	1.72
HLA-B	Major Histocompatability Complex, Class 1, B	0.036	1.7
PRKCD	Protein Kinase C Delta	0.007	1.68
HLA-A	Major Histocompatibility Complex, Class 1, A	0.020	1.66
CD59	CD59 Molecule	0.006	1.65
CASP8	Caspase 8	0.036	1.62
OAS3	2′-5′-oligoadenylate synthetase 3	0.020	1.58
ITGB2	Integrin, Beta-2 Precursor	0.044	1.57
MAP3K1	Mitogen-Activated Protein Kinase Kinase Kinase 1	0.006	1.56
LAMP2	Lysosome-associated membraine protein 2	0.040	1.53
CCL3	Chemokine (C-C Motif) Ligand	0.006	1.52
CDH5	Cadherin 5	0.008	1.51
LILRB1	Leucocyte Immunoglobulin Like Receptor B	0.043	1.51
ICAM1	Intercellular Adhesion Molecule 1	0.011	1.46
MAPK3	Mitogen-Activated Protein Kinase 3	0.015	1.45
CKLF	Chemokine-Like Factor	0.007	1.43
BAX	Bcl-2 like protein 4	0.013	1.41
BST2	Bone Marrow Stromal Cell Antigen 2	0.023	1.41
CD47	Cluster of Differentiation 47	0.016	1.41
CD68	Cluster of Differentiation 68	0.050	1.38
ITGB1	Integrin Subunit Beta 1	0.041	1.35
PSEN1	Presenilin-1	0.049	1.33
NRP1	Neuropilin-1	0.024	1.3
IRF2	Interferon Regulatory Factor 1	0.031	1.27
HCK	Tyrosine-Protein Kinase	0.026	1.26
DDX58	Probable ATP-dependent RNA helicase DDX58	0.003	1.24
ILF3	Interleukin Enhancer Binding Factor 3	0.034	1.19

B Increased expression in low-risk PBC			
MME	Membrane Metallo-Endopeptidase	0.044	4.7
SYT17	Synaptotagmin 17	0.023	3.66
CCL23	Chemokin (C-C motif) ligand 23 (CCL23)	0.019	3.22
IL13RA2	Interleukin-13 Receptor Subunit Alpha-2	0.043	2.84
MAP2K1	Mitogen-Activated Protein Kinase Kinase 1	0.001	2.1
CCL14	C-C Motif Chemokine Ligand 14	0.001	1.93
C9	Complement Component	0.020	1.85
C6	Complement Component 6	0.012	1.8
ARG1	Arginase 1	0.001	1.79
CKLF	Chemokine-Like Factor	0.026	1.78
MIF	Macrophage Migration Inhibitory Factor	0.037	1.78
C8A	Complement Component 8 Alpha Subunit	0.013	1.61
HSD11B1	Hydroxysteroid 11-beta dehydrogenase 1	0.010	1.6
DUSP6	Dual Specificity Phosphatase 6	0.015	1.54
REPS1	RALBP1 Associated Eps Domain Containing 1	0.002	1.54
RORC	RAR-Related Orphan Receptor Gamma	0.038	1.53
C4BPA	Complement Component 4 Binding Protein Alpha	0.006	1.51
MARCO	Macrophage Receptor With Collagenous Structure	0.041	1.51
AMBP	Alpha-1-Microglobulin/Bikunin Precursor	0.001	1.5
C5	Complement Component 5	0.038	1.46
LBP	Lipopolysaccharide Binding Protein	0.027	1.45
C1R	Complement Component 1R	0.004	1.44
ATF1	Activating Transcription Factor 1	0.024	1.42
FEZ1	Fasciculation and Elongation Protein Zeta-1	0.012	1.41
ITCH	Itchy E3 Ubiquitin Protein Ligase	0.039	1.33
C1S	Complement Component 1, S Subcomponent	0.015	1.32
ECSIT	ECSIT Signalling Integrator	0.028	1.3
C1QBP	Complement Component 1, Q Subcomponent Binding Protein	0.015	1.28
ST6GAL1	ST6 beta-galactoside alpha-2,6-sialyltransferase 1	0.013	1.27
MAVS	Mitochondrial Antiviral Signalling Protein	0.018	1.26
RELA	Transcription Factor p65	0.041	1.26
FCGR2B	Fragment of IgG Receptor Iib	0.028	1.23
CKLF	Chemokine-Like Factor	0.026	1.21
